# Enhancing Priority-Setting Decision-Making Process Through Use of Intersectionality for Public Participation

**DOI:** 10.34172/ijhpm.2023.8095

**Published:** 2023-05-21

**Authors:** Anuj Kapilashrami, Donya Razavi, Reza Majdzadeh

**Affiliations:** ^1^School of Health and Social Care, University of Essex, Colchester, UK; ^2^Department of Health, Aging and Society, McMaster University, Hamilton, ON, Canada

## Dear Editor,

 Baltussen et al emphasize in the *conceptual framework of the evidence-informed deliberative processes for legitimate health benefits package design* paper that stakeholder participation is a core element of evidence-informed deliberative processes. The paper introduces seven stakeholder groups which includes patients, public, and carers. They highlight the challenges of stakeholder participation in decision-making processes and assert that such participation can lead to improved legitimacy of decision-making, transparency, and accountability. Stakeholders may accept the trade-off of a fair decision-making process aimed at achieving specific outcomes, even if they may prefer alternative outcomes.^1^ In this letter, we emphasize the need for greater public participation achieved through institutionalizing participation in all stages of priority-setting process, towards empowering communities.

 The neglect of public participation in priority-setting processes, especially involving vulnerable populations, is a concerning trend that continues to persist despite over two decades of literature emphasizing the value-add of public participation in priority-setting processes and the World Health Organization’s (WHO’s) more recent explicit introduction of eight principles for democratic and inclusive decision-making.^2^ Recent experiences in reviewing the design of Essential Package of Health Services in six countries have revealed that only one of the 6 countries, Zanzibar, could demonstrate public participation in priority-setting decision-making.^3,4^ This lack of stakeholder participation in priority-setting processes is previously evidenced by Kapiriri and Razavi comprehensive review of 96 studies spanning from 2000 to 2017, which found that public and vulnerable populations were involved in decision-making processes in only a tiny fraction of the studies, with only 24 studies involving the public and a mere 6 studies involving vulnerable populations.^5^ A more recent systematic review from 2000 to date by Arthur et al identified only 27 studies that involve community actors and other stakeholders in priority-setting and decision-making processes for defining health benefit packages and universal health coverage, health technology assessment, and pharmaceutical coverage. Although a wide range of engagement mechanisms were documented, participation occurred with limited depth of engagement and equity considerations among identified studies.^6^

 In health sector priority-setting and decision-making, powerful stakeholders’ interests often usurp those of less powerful stakeholders.^7^ Such disregard for the importance of community and other stakeholder voice in policy planning, especially lack of participation by the most vulnerable, is unacceptable. Low levels of public participation are consistently reported in the literature, ie, from the inform to empowerment spectrum.^8^ Priority-setting initiatives and engaged stakeholders must prioritize the involvement of the public and vulnerable populations, to ensure that decision-making processes are democratic, inclusive, effective, and considered legitimate by the populations whom these packages are meant to serve.

 The failure to institutionalize public participation in priority-setting has resulted in various instances of failed implementation. For example, a new package in Peru was perceived to have reduced benefits, leading to public backlash. In the Dominican Republic, the lack of communication about the priority-setting criteria and process led to strong opposition from various stakeholders to the updated package.^9^ These cases demonstrate that neglecting public participation can lead to mistrust and opposition, ultimately hindering successful implementation. The case of Thailand, where public involvement has been institutionalized since 2000s,^10^ highlights the potential benefits of involving the public in decision-making processes, such as increased trust and long-term stability.^11^

 The importance of public participation in the priority-setting process cannot be overstated. Without their input and engagement, any resulting changes will likely fall short of achieving meaningful improvements in a country’s healthcare system.^12^ Intersectionality can be useful for identifying vulnerable populations and ensuring their inclusion in the decision-making process. By considering the intersection of various identities and power structures, the decision-making process can better understand different groups’ unique needs and perspectives and ensure that their voices are heard. Intersectionality recognizes the impact of power structures and macro-level discrimination on individual identities and social position such as gender, economic status, and race.^13^ To involve the public in decision-making, we must pay attention to the characteristics of those participating and ensure their needs are met. Moreover, the principles of intersectionality, namely power, reflexivity, and recognizing diverse knowledge can enhance decision-making processes and promote public participation. In examining power, attention is paid to who holds power, and how can power inequities be tackled to address the needs of vulnerable populations. In reflexivity, attention is paid to values and experiences, and in diverse knowledge, their knowledge is utilized in decision-making.^14^

 Overall, while public participation can be an effective conduit for making decision-making processes more effective, democratic, and sustainable, foregrounding the principle of intersectionality can be central to ensuring that the needs of vulnerable populations are met, and their voices are heard.

## Ethical issues

 Not applicable.

## Competing interests

 Authors declare that they have no competing interests.
